# Primary small cell neuroendocrine carcinoma in the nasal cavity: A CARE-compliant case report

**DOI:** 10.1097/MD.0000000000027136

**Published:** 2021-09-03

**Authors:** Li-Yu Chen, Shih-Lun Chang, Wen-Ying Lee

**Affiliations:** a Department of Otorhinolaryngology, Chi Mei Medical Center, Tainan, Taiwan; b Department of Optometry, Chung Hwa University of Medical Technology, Tainan, Taiwan; c Department of Cytopathology, Chi Mei Medical Center, Tainan, Taiwan.

**Keywords:** nasal cavity, neuroendocrine carcinoma, paranasal sinuses, small cell carcinoma

## Abstract

**Rationale:**

Small cell neuroendocrine carcinoma of the nasal cavity and paranasal sinuses is a rare but aggressive neoplasm with a poor prognosis and a strong propensity for regional recurrence and distant metastasis. Diagnosis is challenging and relies on immunohistochemical study. Treatment includes surgical resection, radiation therapy, chemotherapy, or a combination of these modalities. However, the optimal therapeutic strategy is still controversial. Due to its rarity, the complexity of the histological diagnosis, and the variety of the treatment regimens, we presented a case of primary small cell neuroendocrine carcinoma in the nasal cavity with description of the clinical manifestation, pathology features, and our treatment regimen.

**Patient concerns:**

An 82-year-old female patient with hypertension presented with right epistaxis on and off with nasal obstruction for several days.

**Diagnosis:**

An exophytic mass over the posterior end of the right inferior turbinate was found on nasopharyngoscope. Biopsy was done and the pathology confirmed small cell carcinoma, strongly positive for cytokeratin (AE1/AE3) and insulinoma-associated protein 1 (INSM-1), scatteredly positive for chromogranin A, synaptophysin and CD56. The final diagnosis was small cell neuroendocrine carcinoma of right nasal cavity, pT1N0M0, stage I.

**Interventions:**

The patient underwent wide excision of right intra-nasal tumor and post-operative radiotherapy with a dose of 6600 cGy in 33 fractions.

**Outcomes:**

No local recurrence or distant metastasis was noted during the 12 months of follow-up.

**Lessons:**

Multimodality treatment remains the most common therapeutic strategy, although no proven algorithm has been established due to the rarity of this disease. Further investigation is needed for providing evidence to standardize the treatment protocol.

## 1 Introduction

Small cell neuroendocrine carcinoma in the nasal cavity and paranasal sinuses is a rare neoplasm with aggressive clinical behavior and poor prognosis.^[1,2]^ Its morphology is similar to those occurring in pulmonary or other extrapulmonary sites. There is a male gender propensity and usually aged 24 to 79 years old with a mean age of about 50 years old.^[1,3,4]^ The nasal cavity is the most common location followed by the ethmoid sinus and the maxillary sinus. Nasal obstruction and epistaxis are the most common presenting symptoms.^[1]^ Diagnosis is challenging and relies on immunohistochemical study. Treatment include surgical resection, radiotherapy, chemotherapy, or a combination of these modalities. But there is no consensus on the optimal treatment.^[2–5]^ Due to its rarity, the complexity of the histological diagnosis, and the variety of the treatment regimens, we present a case of primary small cell neuroendocrine carcinoma in the nasal cavity with description of the clinical manifestation, pathology features, and our treatment regimen.

## 2 Case report

An 82-year-old female patient presented with right epistaxis on and off for several days, accompanied by the right nasal obstruction. Symptoms such as purulent rhinorrhea, post-nasal dripping, or sneezing were not observed. She denied facial trauma, nose picking or the presence of a foreign body in the nasal cavity. Her medical history included hypertension under regular medication control without any anticoagulant or anti-platelet agent use, and she had no surgical history before. She had no exposure to cigarette, alcohol, or betel nuts. There was neither history of ear, nose, and throat problems nor any family history of such. She was brought to our Otolaryngology out-patient department for further evaluation.

**Figure 1:**
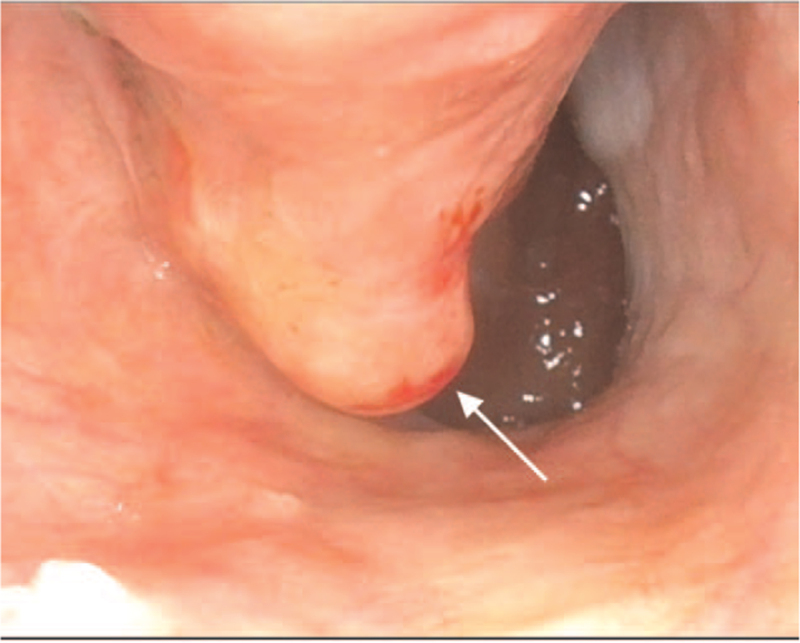
Nasopharyngoscopy showed an exophytic mass (arrow) with a smooth mucosal surface over the posterior end of the right inferior turbinate.

**Figure 2:**
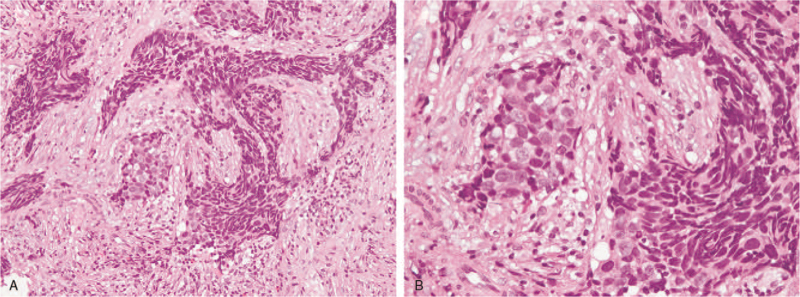
Infiltration by sheets of tumor cells with high nuclear cytoplasmic ratio, dense chromatin and inconspicuous nucleoli. Marked crushing artifact and nuclear molding is present. These features are characteristic for small cell carcinoma. (A, Hematoxylin and eosin, 200x. B, Hematoxylin and eosin, 400x.).

**Figure 3:**
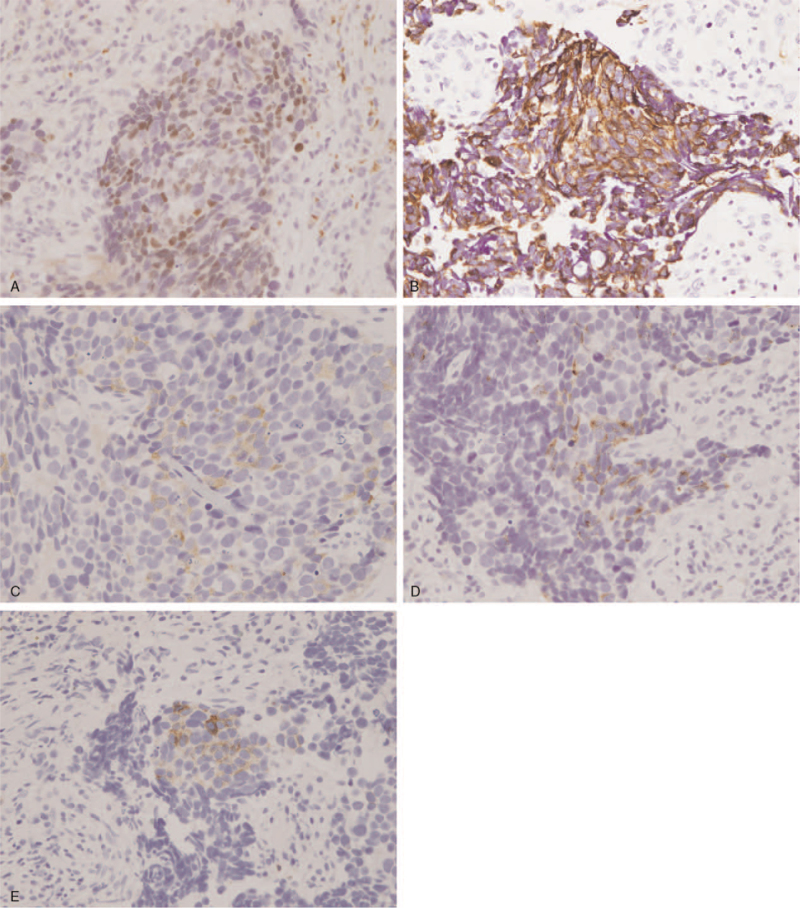
Immunohistochemical study showed the tumor cells are strongly positive for (A) insulinoma-associated protein 1 (INSM-1) and (B) cytokeratin (AE1/AE3). And it is scatteredly positive for (C) chromogranin A, (D) synaptophysin, and (E) CD56.

No cervical lymphadenopathy was noted on physical examination. Nasopharyngoscopy found that the bleeder arise from the right inferior turbinate. A bulging mass over the posterior end of the right inferior turbinate was revealed after hemostasis (Fig. 1). The mass was exophytic with a smooth mucosal surface, and inverted papilloma or polyp was suspected initially. Polypectomy of right nasal cavity was performed and the pathology report confirmed the finding of small cell neuroendocrine carcinoma (Fig. 2). Immunohistochemical analysis showed that the tumor cells are strongly positive for cytokeratin (AE1/AE3) and insulinoma-associated protein 1 (INSM-1), and scatteredly positive for chromogranin A, synaptophysin, and CD56, supporting the diagnosis (Fig. 3).

**Figure 4:**
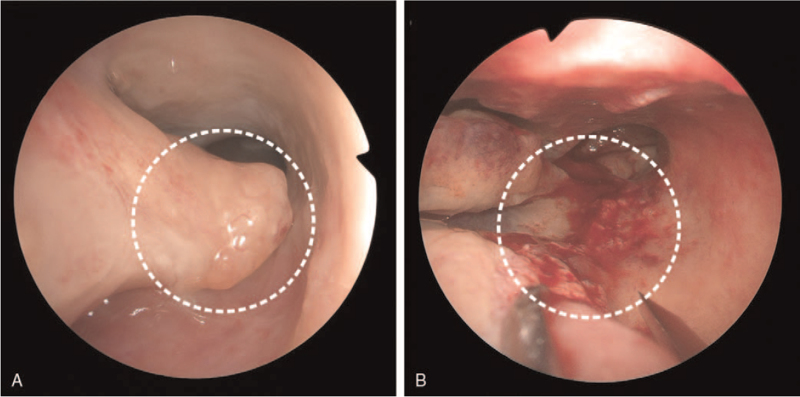
(A) Sinuscopy during operation of wide excision showed an exophytic mass over posterior end of right inferior turbinate. (B) The tumor was completely excised with nearly no residual inferior turbinate.

A series of examinations for tumor staging including chest x-ray, head and neck magnetic resonance imaging with contrast, abdominal sonography, esophagogastroduodenoscopy, and whole body bone scan were arranged. The head and neck magnetic resonance imaging with contrast showed no definite abnormal enhancing mass lesion at operation bed and no definite neck nodal metastasis, stage TxN0. Neither second primary tumor, lymph nodes metastasis nor distant metastasis was found. Wide excision of the right intra-nasal tumor was performed. (Fig. 4.) The largest tumor size was 1 cm in dimension and the tumor invasion confined in the inferior turbinate with deep surgical margin involvement. There is no venous, lymphatic, or perineurial invasion. The final diagnosis was small cell neuroendocrine carcinoma of right nasal cavity, pT1N0M0, stage I.

The patient then received postoperative external beam radiation therapy with a dose of 6600 cGy in 33 fractions. The patient had regular follow-up at the otolaryngology and the radiation oncology outpatient department with a follow-up period of 12 months. There was no local tumor recurrence noted in the endoscopy during postoperative monthly follow-up. Neck CT which was arranged 3 months later after completion of radiation therapy also revealed no local recurrence or neck nodal metastasis.

## 3 Discussion

Small cell neuroendocrine carcinoma (SCNEC) in the extra-pulmonary sites is rare, accounting for 2.5 to 5 percent of SCNEC,^[7,8]^ and has poor prognosis with a 13% five-year survival rate.^[6]^ Small cell carcinoma in head and neck only accounts for 10 to 15 percent of extra-pulmonary SCNEC (approximately 0.3% of all SCNEC) with lower five-year survival rates.^[1,7]^ The most common anatomic site is the larynx followed by the nasal cavity and paranasal sinuses. And the patients with the nasal cavity and paranasal sinuses primaries have the best survival rate.^[9]^

The patients with SCNEC of the nasal cavity and paranasal sinuses were usually aged 24 to 79 years old with a mean age of about 50 years. Few studies indicated a male gender propensity and a possible association with smoking. Male preponderance was supported by a systemic review, but association with smoking could not be proven due to tobacco exposure was not documented in enough studies for significant analysis. Most of the studies lacked data on racial diversity that further investigation is needed for racial predilection.^[1,3–5]^

SCNEC of the nasal cavity and paranasal sinuses present a variety of clinical manifestation. The most common presenting symptoms are nasal obstruction and epistaxis. Other symptoms include facial swelling, facial pain or pressure, neck lymphadenopathy, change of vision, proptosis, cranial nerve disturbance, and headache. The most common primary sites are the nasal cavity and nasal septum followed by the ethmoid sinus and then maxillary sinus. Rarely, it may arise from the sphenoid sinus and frontal sinus.^[1–5]^

The diversity and non-specificity of the presenting symptoms make the diagnosis of SCNEC in the nasal cavity and paranasal sinuses particularly challenging and relies on histopathology demonstration. The morphology is similar to those occurring in the pulmonary or extra-pulmonary sites. Morphological features including densely cellular tumor cells arranged in sheets, cords, or ribbons with a high nuclear-to-cytoplasmic ratio, dense chromatin and a high mitotic rate. Immunohistochemical markers are useful to establish the diagnosis. Neuronspecific enolase (NSE), synaptophysin, and chromogranin tend to be positive in most of the cases.^[1,2,4]^ Other markers include CD56, Cytokeratin AE1/AE3, and Insulinoma-associated protein 1 (INSM-1).^[3,5,10]^ The expression is variable. In our case, the tumor cells are strongly positive for cytokeratin (AE1/AE3) and INSM1, and scatteredly positive for chromogranin A, synaptophysin, and CD56.

No proven algorithm has been established for the treatment of SCNEC in the nasal cavity and paranasal sinuses.^[1,8]^ There is variability in treatment modality including surgery, radiotherapy, chemotherapy, and combinations.^[1,4,8]^ The optimal therapeutic strategy is still controversial. Most of the literature agreed that the management may be similar to the pulmonary small cell carcinoma.^[7–9]^ Radical surgery and definite radiotherapy may be chosen in early or limited disease, while chemotherapy might be preserved for locally advanced disease.^[7–9]^ The most common regimens of chemotherapy are the combination of cisplatin and etoposide.^[4,8]^ Muhammad et al. had reported the radiation dose ranging from 50 to 60 Gy with good local control as compared to 60 to 70 Gy which was standard protocol in other centers.^[3]^ A few studies suggest surgery with postoperative chemoradiotherapy might reduce the risk of regional recurrence and distant metastasis. However, no therapeutic recommendation could be determined yet.

The prognosis of SCNEC of the nasal cavity and paranasal sinuses is poor due to the high risk of local recurrence and distant metastasis despite multimodality treatment. The median time to recurrence is 9 months according to a recent systemic review.^[1]^ Furthermore, most of the patients presented with advance stage (AJCC stage III and stage IV) when diagnosed. The majority of these patients were alive with disease or died of disease. Only a few people could be alive without evidence of disease at follow-up.^[1]^

According to experience of the previous literature, considering the early stage with limited disease and the old age of the patient, we choose surgical resection with postoperative radiation therapy as our treatment regimen. The dose of radiation therapy was 6600 cGy in 33 fractions similarly with the standard protocol in some centers, which was mentioned in previous literature.^[3]^ The patient was diagnosed in early stage, which we could foresee the better outcome. There is no local recurrence, regional or distant metastasis during a follow-up period of 12 months.

## 4 Conclusion

Small cell neuroendocrine carcinoma of the nasal cavity and paranasal sinuses is a rare but aggressive neoplasm with a poor prognosis and a strong propensity for local recurrence and distant metastasis. Multimodality treatment remains the most common strategy, although no proven algorithm has been established due to the rarity of this disease. Further investigation is needed for providing evidence to standardize the treatment protocol.

## Author contributions

**Resources:** Shih-Lun Chang, Wen-Ying Lee.

**Supervision:** Shih-Lun Chang.

**Writing – original draft:** Li-Yu Chen.

**Writing – review & editing:** Li-Yu Chen, Shih-Lun Chang.
